# Indikation und präoperative Planung zur bariatrischen Operation

**DOI:** 10.1007/s00508-023-02271-8

**Published:** 2023-10-11

**Authors:** Daniel Moritz Felsenreich, Andrea Malzner, Marlies Eichner, Elisabeth Hoelbing, Alexander Moosbrugger, Philipp Beckerhinn, Gerhard Prager, Johanna Maria Brix, Bianca-Karla Itariu

**Affiliations:** 1 Klinische Abteilung für Viszeralchirurgie, Universitätsklinik für Allgemeinchirurgie, Medizinische Universität Wien, Wien, Österreich; 2 Abteilung für Innere Medizin I, Gastroenterologie und Hepatologie, Rheumatologie, Endokrinologie und Diabetologie, Klinikum Wels-Grieskirchen, Wels, Österreich; 3 3. Medizinische Abteilung mit Stoffwechselerkrankungen und Nephrologie, Klinik Hietzing, Wien, Österreich; 4 Landeskrankenhaus Hochsteiermark, Standort Leoben, Leoben, Österreich; 5 Abteilung für Innere Medizin II, Krankenhaus der Barmherzigen Brüder, Graz, Österreich; 6 Abteilung für Chirurgie, Landesklinikum Hollabrunn, Hollabrunn, Österreich; 7 1. Medizinische Abteilung mit Diabetologie, Endokrinologie und Nephrologie, Klinik Landstraße, Wien, Österreich; 8 Karl Landsteiner Institut für Adipositas und Stoffwechselerkrankungen, Klinik Landstraße, Wien, Österreich; 9 Klinische Abteilung für Endokrinologie und Stoffwechsel, Universitätsklinik für Innere Medizin III, Medizinische Universität Wien, Wien, Österreich

**Keywords:** Adipositas, Bariatrische Chirurgie, Präoperative Untersuchung, Präoperatives Vorgehen, Fast-Track-Indikation, Obesity, Bariatric surgery, Preoperative examinations, Preoperative approach, Fast track indications

## Abstract

Dieser Text stellt eine Handlungsanleitung dar, welche im Konsens mehrerer österreichischer mit der Therapie von Adipositas assoziierter FachärztInnen erstellt wurde. Dabei wurde neben aktueller Literatur und bestehenden Guidelines auch zwischen Machbarkeit von ärztlicher Seite und Zumutbarkeit gegenüber den PatientInnen abgewogen. Besonderer Wert wurde auf Vereinfachung der präoperativen Abklärung bei maximaler Sicherheit gelegt. Daher stellt dieser Text keinen Anspruch auf Vollständigkeit in allen Bereichen.

## Abstract (en)

These clinical practice guidelines represent the consensus opinion of a group of Austrian specialist physicians associated with the treatment of obesity. The recommendations incorporate the current literature and guidelines and aim to balance both procedural feasibility and patient acceptance and adherence. Special emphasis was placed on simplification of the preoperative clarification and maximum patient safety. Therefore, this article makes no claim to be complete in all fields.

## Indikationen zur bariatrischen Operation

Laut den aktuellen internationalen ASMBS/IFSO-Leitlinien (American Society for Metabolic and Bariatric Surgery/International Federation for the Surgery of Obesity and Metabolic Disorders) (2022) kann ein bariatrischer/metabolischer Eingriff unabhängig von Begleiterkrankungen ab einem Body Mass Index (BMI) ≥ 35 kg/m^2^ durchgeführt werden. Bei Menschen mit metabolischer Erkrankung und einem BMI zwischen 30 kg/m^2^ und 34,9 kg/m^2^ sollte eine metabolische Operation in Erwägung gezogen werden [1].

Die Guidelines der European Association for the Study of Obesity (2015) [2], die deutsche S3-Leitlinie (2018; in Überarbeitung) [3] und die aktuellen Leitlinien der Adipositas-Gesellschaften in Kanada (2020) [4] und in Irland (2022) [5] empfehlen bariatrisch-chirurgische Eingriffe ab einem BMI ≥ 40 kg/m^2^ bzw. ab einem BMI ≥ 35 kg/m^2^, wenn zumindest eine Adipositas-assoziierte Komorbidität vorliegt.

Die Österreichische Adipositas Gesellschaft empfiehlt einen bariatrisch-chirurgischen Eingriff bei Erwachsenen derzeit, wenn ein suffizienter Gewichtsverlust durch konservative Therapiemaßnahmen nicht erreicht werden kann, bei Personen mit einem BMI ≥ 35 kg/m^2^, deren Gesundheit durch eine Gewichtsreduktion günstig beeinflusst werden kann. Bei Personen mit einem BMI ≥ 30 kg/m^2^ und Adipositas-assoziierten Komorbiditäten kann eine bariatrische Operation in Erwägung gezogen werden (Tab. 1, Empfehlungen 1.1; [6]).

**Table 1:** 

**1.1. Indikationsstellung**
Chirurgische Eingriffe sind indiziert nach vorangegangenen, frustran verlaufenen konservativen Therapieversuchen [6] – bei BMI ≥ 40 kg/m^2^ oder – bei BMI ≥ 35 kg/m^2^ mit Begleiterkrankungen/Risikofaktoren, die durch eine Gewichtsreduktion günstig beeinflusst werden können – Bei Personen mit einem BMI ≥ 30 kg/m^2^ und Adipositas-assoziierten Komorbiditäten kann eine bariatrische Operation in Erwägung gezogen werden
Die Indikation zur bariatrischen Chirurgie soll multidisziplinär gestellt werden
Das multidisziplinäre Team zur Betreuung der Patienten soll alle Aspekte der prä-, peri- und postoperativen Betreuung abdecken und idealerweise folgende Berufsgruppen umfassen: – ÄrztIn für Allgemeinmedizin – ÄrztIn für Innere Medizin mit Schwerpunkt Endokrinologie – ÄrztIn für Chirurgie – AnästhesistIn – VertreterIn von Gesundheitsberufen mit einer PSY-Kompetenz (z. B. klinische PsychologInnen bzw. GesundheitspsychologInnen, PsychotherapeutInnen oder PsychiaterInnen) – DiätologInnen – Pflegefachkraft (gehobener Dienst) – SozialarbeiterIn (bei Bedarf)
Kontraindikationen für einen bariatrischen Eingriff sind: – konsumierende und neoplastische Erkrankungen in den vorangegangenen 5 Jahren – lebensverkürzende Erkrankungen, wie z. B. dekompensierte Leberzirrhose – andere schwer gesundheitlich einschränkende Erkrankungen, welche sich durch den postoperativen katabolen Stoffwechsel verschlechtern können – aktive Substanzabhängigkeit (Drogen, Alkohol) – instabile psychopathologische Zustände oder schwere Depression – unbehandelte Bulimia nervosa – schwere psychiatrische Erkrankung (Ausnahme: reaktive Depression aufgrund der Adipositas) – mangelnde Fähigkeit oder mangelnde Bereitschaft, an einem langfristigen medizinischen Follow-up teilzunehmen
**1.2. Präoperatives Vorgehen**
Bestehende gewichtsreduzierende Programme sollen präoperativ beibehalten werden
Die Therapie von Begleiterkrankungen soll optimiert werden (z. B. Substitution einer Hypothyreose, Optimierung der Blutzucker- bzw. Blutdruckeinstellung etc.)
Eine umfassende, folgende Punkte beinhaltende präoperative Evaluierung soll erfolgen: – Nutzen-Schaden-Analyse – Bestimmung des Operationsrisikos, präoperative Untersuchung zur Abklärung der allgemeinen Operationstauglichkeit (wie bei anderen größeren abdominellen Operationen) – Routinelabor (inklusive Nüchternblutzucker, HbA_1c_, Lipide, Nierenfunktion, Leberwerte [inklusive Cholinesterase], proBNP, Gesamteiweiß, Albumin, Harnanalyse, Gerinnung, Blutgruppe, Blutbild, Schilddrüsenwerte, Kalzium, Vitamin D, Parathormon) – Nährstoffscreening (Eisenstatus, Vitamin B_12_, Folsäure, Vitamin D; optional: Vitamin A, Vitamin E, Zink, Kupfer, Selen) – kardiopulmonale Untersuchung (inklusive Schlafapnoesyndrom) – gastrointestinale Untersuchung (Gastroskopie, *Helicobacter-pylori*-Screening, Oberbauch-Sonographie [Leber, Gallenblase]) – endokrinologische Untersuchung – Knochendichtemessung – Ernährungsanamnese – Erhebung von Essstörungen (z. B. Binge-Eating), um eine entsprechende präoperative Verhaltenstherapie einzuleiten – Anamnese des psychosozialen Verhaltens
Psychosoziale Unterstützung
Zu folgenden Punkten sollen die PatientInnen umfassend aufgeklärt und beraten werden: – die verschiedenen Operationsverfahren, der jeweils erwartbare Gewichtsverlust und die Operationsrisiken – die notwendige postoperative Ernährungsumstellung – die Notwendigkeit einer langfristigen Nachsorge mit regelmäßigen Kontrolluntersuchungen – lebenslange Vitaminsubstitution Sind die PatientInnen damit nicht einverstanden, soll der Eingriff nicht durchgeführt werden

## Kontraindikationen zur bariatrischen Operation

Kontraindikationen zu Adipositas-chirurgischen Eingriffen sind einerseits mangelnde Compliance zur lebenslangen täglichen Vitamineinnahme und zur lebenslangen Nachsorge sowie bestehende Schwangerschaft, aktiver Alkohol- und Drogenabusus, Karzinomanamnese in den vorangegangenen 5 Jahren und mehrere schwere psychiatrische Erkrankungen (Tab. 1, Empfehlungen 1.1). Keine Kontraindikation stellen reaktive Depressionen dar, welche sich auf das Gewicht zurückführen lassen [7].

## Spezielle Indikationen zur bariatrischen Operation

### PatientInnen mit Adipositas im höheren Alter

Prinzipiell gibt es keine Altersobergrenze, allerdings sollte bei PatientInnen höheren Alters eine Operation ohne bzw. mit nur schwacher malabsorptiver Komponente gewählt werden (z. B. Sleeve-Gastrektomie) [8].

### Kinder und Jugendliche mit Adipositas

In der Gruppe der Jugendlichen mit extremer Adipositas ist es sinnvoll, frühzeitig an eine Operation zu denken, da die Chance der Remission von Komorbiditäten wie Typ-2-Diabetes mellitus (T2DM) höher ist, je kürzer diese bestehen [9]. Allerdings sind Jugendliche als Personengruppe besonders zu schützen, wodurch spezifische strenge Kriterien für die Indikationsstellung bestehen und nur bariatrische Zentren in Zusammenarbeit mit PädiaterInnen, InternistInnen und ChirurgInnen mit viel Erfahrung diese Eingriffe vornehmen sollten [10].

Die American Academy of Pediatrics (2023) [11] und die ASMBS [1] empfehlen eine Evaluierung der Indikation zur bariatrischen Operation für Kinder und Jugendliche mit einem BMI > 120 % der 95. Perzentile (Adipositas, Klasse II) mit relevanten Komplikationen oder aber bei einem BMI > 140 % der 95. Perzentile (Adipositas, Klasse III). Nach dem Nationalen Konzept zur Therapie von Übergewicht und Adipositas im Kindes- und Jugendalter in Österreich (2022) ist die bariatrische Chirurgie für ausgewählte Jugendliche als Therapieoption in Betracht zu ziehen, wenn bereits alle konservativen Behandlungsoptionen ausgeschöpft wurden. Bariatrisch-chirurgische Eingriffe sollten ausschließlich in Zentren mit ausgewiesener Expertise angeboten werden [12].

### PatientInnen mit *NASH*, Leberfibrose bzw. Leberzirrhose

Da Adipositas bei PatientInnen mit Leberzirrhose ein Hauptrisikofaktor für Dekompensation, Pfortaderthrombose, hepatozelluläres Karzinom (HCC) und die Entwicklung einer akuten Komplikation ist, sollte die Gewichtsabnahme ein wichtiges Therapieziel sein [13]. Prinzipiell ist die Leberfunktion nach Adipositaschirurgie verbessert, kann bei bereits vorgeschädigter Leber durch den vermehrten Anfall von Abbauprodukten bei starkem Gewichtsverlust aber auch weiter abnehmen. Eine bariatrische Operation sollte daher interdisziplinär evaluiert und ggf. eher ein Eingriff ohne malabsorptive Komponente (z. B. Sleeve-Gastrektomie) in einem Exzellenz-Zentrum gewählt werden [14].

Die Entscheidung zur Operation hängt vom Grad der Lebererkrankung ab; dies umfasst insbesondere das Vorhandensein und den Grad von Sarkopenie, Ödemen/Aszites und portaler Hypertension, das Vorliegen einer kompensierten oder dekompensierten Zirrhose, das PatientInnen-Alter und die Eignung für eine Lebertransplantation.

Bei PatientInnen mit kompensierter Leberzirrhose sollte eine Operation evaluiert werden, um das Risiko für ein HCC zu senken bzw. um die Überlebenswahrscheinlichkeit zu steigern [13]. Des Weiteren ist es wichtig, im Vorhinein den Alkoholkonsum zu evaluieren, da postoperativ ein ungesunder Alkoholkonsum zunehmen und die Lebererkrankung verschlimmern kann. Auch müssen die PatientInnen darüber aufgeklärt werden, dass bei intraoperativ hochgradigem Zirrhoseverdacht (beruhend auf den makroskopischen Befunden) auf eine Operationsmethode ohne malabsorptive Komponente gewechselt wird. Jedenfalls sollten präoperativ ein Fib‑4 errechnet und ein Ultraschall des Abdomens bzw. (je nach Verfügbarkeit) auch ein FibroScan (Echosens SA, Paris, Frankreich) durchgeführt werden, um eine Graduierung – Fettleber, nichtalkoholische Steatohepatitis (NASH), Leberzirrhose – vorzunehmen [15]. Bei bioptisch gesicherter präoperativer NASH scheint die Adipositaschirurgie im Vergleich zur nichtchirurgischen Behandlung mit einem signifikant niedrigeren Risiko für schwerwiegende unerwünschte Leberschäden und anderen Endpunkten verbunden zu sein [16]. Die Adipositaschirurgie führt zudem zu einer Reduktion des Progressionsrisikos von NASH zur Leberzirrhose um 88 % [16]. Intraoperativ sollte bei diesen PatientInnen eine Leberbiopsie durchgeführt werden, um einen Vergleichswert für eine später durchgeführte Diagnostik zu haben.

Bei dekompensierter Leberzirrhose ist die einzige akzeptable Option derzeit die bariatrische Chirurgie, die gleichzeitig mit oder nach einer Lebertransplantation durchgeführt wird [13].

### Patientinnen mit Kinderwunsch

Ein Kinderwunsch stellt keine Kontraindikation dar, allerdings sollten die Patientinnen aufgeklärt werden, dass im ersten Jahr postoperativ keine Schwangerschaft geplant werden soll, da ein initialer Gewichtsverlust nach der Operation komplett abgeschlossen sein sollte [17]. Bei frühzeitiger Schwangerschaft nach einer bariatrischen Intervention besteht einerseits ein erhöhtes Risiko von inneren Hernien und andererseits das Risiko, dass sich ein kataboler Stoffwechsel (oder relative Ernährungsmängel) negativ auf die Entwicklung des Kindes auswirkt [18]. Schwangere Patientinnen nach bariatrischer Operation sollten engmaschig kontrolliert werden, um Mikronährstoffmängel zu evaluieren und rechtzeitig zu substituieren und um das Risiko von „Small for gestational age“-Neugeborenen zu minimieren [19, 20].

### Rauchende PatientInnen

Zigarettenrauchen stellt keine Kontraindikation zur bariatrischen Operation dar, allerdings sollten die PatientInnen zum Rauchstopp motiviert werden, da postoperativ ein erheblich höheres Risiko eines Anastomosenulkus besteht. Rauchende PatientInnen sollten außerdem für die Dauer eines Jahres postoperativ eine Protonenpumpeninhibitorentherapie erhalten [21].

### Fast-Track-Indikation

Allgemein beträgt die Zeit ab Indikationsstellung bzw. Einwilligung zur Operation bis zum Eingriff mehrere Monate. Bei bestimmten PatientInnen mit bereits vorhandenen Komplikationen (Tab. 2) wird ein beschleunigter Zugang zur bariatrisch-metabolischen Chirurgie empfohlen, um den Schweregrad der Erkrankung rechtzeitig reduzieren zu können [22]. In jedem Fall muss eine gründliche präoperative Abklärung erfolgen.

**Table 2:** 

Indikation
PatientInnen mit Typ-2-Diabetes (T2DM) und einem HbA_1c_ ≥ 8 %, – die 2 oder mehr orale Antidiabetika (bzw. GLP-1-Rezeptoragonisten) erhalten und/oder Insulintherapie benötigen, – die eine kardiovaskuläre Vorerkrankung oder eine nichtalkoholische Fettlebererkrankung (NAFLD) oder ≥ 2 Stoffwechselstörungen, die das kardiovaskuläre Risiko erhöhen, aufweisen, – die eine persistierende Albuminurie/Proteinurie oder chronische Nierenerkrankung (CKD; Grad 3–4) haben, – die eine Diabetesdauer von ≥ 5 Jahren aufweisen
PatientInnen mit Adipositas und mehr als 2 Stoffwechselkomplikationen (außer T2DM), welche das kardiovaskuläre Risiko erhöhen (z. B. NAFLD oder arterielle Hypertonie)
PatientInnen mit Adipositas und Herzinsuffizienz (AHA-Klasse C)
PatientInnen mit CKD, Grad 3–4
PatientInnen, die eine Indikation zur Organtransplantation haben, mit einem BMI oberhalb der Grenze, die eine Kontraindikation zur Transplantation darstellt
PatientInnen mit Indikation zu anderen chirurgischen Eingriffen, wie z. B. Koronarbypass oder anderen herzchirurgischen Eingriffen, Knie oder Hüftendoprothesen, Wirbelsäulenchirurgie oder Infertilität aufgrund der Adipositas
PatientInnen mit BMI ≥ 50 kg/m^2^

## Wer stellt die Indikation zur bariatrischen Operation?

PatientInnen, bei denen von internistischer, chirurgischer oder allgemeinmedizinischer Seite die Indikation zur bariatrischen-chirurgischen Therapie gestellt wurde, werden in ein bariatrisches Zentrum zugewiesen. Alle PatientInnen sollten präoperativ durch ein multidisziplinäres Team mit internistischer, chirurgischer, ernährungsmedizinischer und psychiatrischer Expertise evaluiert werden. Nach Einholen und sorgfältiger Überprüfung aller Befunde stellt das bariatrisch-chirurgische Zentrum die Indikation zur Operation (und die „surgical readiness“) fest. Nach Einwilligung der PatientInnen wird die Operation terminiert. Dabei wird auch, gemeinsam mit der/m ChirurgIn, die für die individuelle Situation beste Operationsform gewählt.

## Gutachten vor Durchführung einer bariatrischen Operation

### Psychologisches Gutachten

Zur Evaluierung der ausreichenden Compliance der PatientInnen, regelmäßige Kontrollbesuche und eine lebenslange Vitamineinnahme einzuhalten, muss ein psychologisches Gutachten durchgeführt werden (Tab. 3). Dabei werden auch Erkrankungen des psychischen Formenkreises, Essstörungen, Depressionen, Suchterkrankungen usw. abgeklärt, und es wird evaluiert, ob in der ersten Zeit nach der Operation eine weiterführende psychologische Begleitung notwendig ist.

**Table 3:** 

Untersuchungen und Gutachten
Komplette klinische Anamnese
Internistische/chirurgische Abklärung: – Blutdruck, optional: Echokardiographie – Lungenröntgen, optional: Lungenfunktion – Ultraschall des Abdomens (Leber, Gallenblase) – Gastroskopie (Abklärung einer Infektion mit *H. pylori*, Zwerchfellhernien) – optional: 24-h-pH-Metrie und Manometrie (Motilitätsstörung, Refluxerkrankung) – optional: Knochendichtemessung – Abklärung eines Cushing-Syndroms (bei klinischem Verdacht)
*Labor:* Blutbild, Differenzialblutbild, Gerinnung, Elektrolyte, Albumin, Gesamteiweiß, Kreatinin, BUN, Harnsäure, GOT, GPT, GGT, AP, Bilirubin, Kreatininkinase, Nüchternblutzucker, HbA_1c_, Triglyzeride, Gesamt‑, HDL- und LDL-Cholesterin, Lp(a), Eisenstatus, Folsäure, 25 Hydroxy-Vitamin‑D, Vitamin B_12_, C‑reaktives Protein (CRP), B‑Typ-natriuretisches Peptid (BNP), Thyreotropin (TSH), freies Thyroxin (fT4), Parathormon
Psychologisches Gutachten: Compliance, Erkrankungen des psychischen Formenkreises, Essstörungen, Suchtverhalten
Diätologisches Gutachten und Schulung

Bei psychischen Erkrankungen mit entsprechender Medikamenteneinnahme sollte ein/e betreuende/r FachärztIn für Psychiatrie in die chirurgische Behandlungsplanung eingebunden werden.

### Diätologisches Gutachten

Im Rahmen des diätologischen Gutachtens werden die PatientInnen im Vorfeld der bariatrischen Operation auf die neue Essenssituation nach dem Eingriff vorbereitet (Tab. 3). Dabei wird auch evaluiert, ob bereits suffizient auf konservative Art und Weise (unter professioneller Anleitung) versucht wurde, das Gewicht zu reduzieren.

Besonders PatientInnen mit einem BMI von ≥ 50 kg/m^2^ wurde empfohlen, vor der Operation zunächst mithilfe von diätetischen Maßnahmen und medikamentöser Therapie („Leberfasten“, kohlehydratarme Diät, GLP-1-Rezeptoragonisten) 5–10 % des Körpergewichts zu verlieren, um die Größe der Leber und somit das Operationsrisiko zu reduzieren. Die Empfehlung wird mittlerweile durch eine Reihe von Studiendaten gestützt [23–25]. Zusätzlich zum Gutachten ist eine diätologische Schulung bzw. Anleitung für die Ernährungsumstellung nach der Operation sinnvoll.

## Weitere Untersuchungen vor bariatrischer Operation

### Internistische Abklärung

Präoperativ sollte eine komplette Anamnese und internistische Untersuchung inklusive Blutdruck- und Blutzuckermessung durchgeführt werden (Tab. 3). Außerdem sollte die Klassifizierung der Adipositas nach „Edmonton-Stadium“ [26] vorgenommen werden, da das perioperative Risiko bei Adipositas im Stadium 3 und 4 deutlich höher ist. Nicht oder unzureichend kontrollierter T2DM sollte im Vorfeld der Operation den Behandlungsrichtlinien entsprechend eingestellt werden. Bei BMI > 70 kg/m^2^ sollte eine Operation gründlich und multidisziplinär besprochen (Adipositas-Boards; s. unten) und jedenfalls in einem Zentrum mit ausgewiesener Expertise durchgeführt werden. Des Weiteren wird eine apparative Untersuchung mittels Lungenfunktion, Echokardiographie, Lungenröntgen und EKG empfohlen, um mögliche Pathologien frühzeitig auszuschließen. Im Rahmen der internistischen Freigabe sollte die/der PatientIn außerdem anästhesiologisch vorgestellt werden.

### Laborkontrollen

Präoperativ wird ein Standardlaborpanel empfohlen, bestehend aus Blutbild und Differenzialblutbild, Gerinnung, Serumchemie inklusive Elektrolyten, Nieren‑, Leber- und Schilddrüsenwerten sowie Blutzucker und Blutfetten (Tab. 3).

### Abklärung eines Cushing-Syndroms

In vielen österreichischen Zentren wird ein Hemmtest mit Dexamethason 1 mg (oder ähnliche Suppressionstests) durchgeführt, jedoch zeigt die Datenlage, dass die Prävalenz von Cushing-Syndrom in einem spezialisierten bariatrischen Zentrum mit 0,6 % sehr niedrig ist [27]. Daten aus der Türkei deuten auf eine Hyperkortisolismusprävalenz von bis zu 5,4 %, wobei die autonome Kortisolüberproduktion mit 4 % führend ist. Klinische Augenmerke bei den PatientInnen mit Hyperkortisolismus waren Adipositas, Klasse I, arterielle Hypertonie, unkontrollierter T2DM und Alter ≥ 50 Jahre [28]. Wir empfehlen somit, den Dexamethason-Hemmtest vor bariatrischer Operation nicht routinemäßig bei allen PatientInnen durchzuführen, sondern nur bei klinischem Verdacht auf Hyperkortisolismus, bei unkontrolliertem T2DM oder bei unkontrollierter arterieller Hypertonie mit/ohne Hypokaliämie. Dazu ist das klinische Erscheinungsbild bei Statusuntersuchung zu erheben.

### Ultraschall des Abdomens

Zur Beurteilung von abdominellen Auffälligkeiten besonders in der Leber sollte ein Ultraschall des Abdomens durchgeführt werden. Dabei können die Leberkonstitution (z. B. Steatose, Fibrose, Zirrhose) oder allfällige Läsionen evaluiert werden. Des Weiteren kann die Gallenblase auf Steine untersucht werden.

### Gastroskopie und *Helicobacter*-Eradikation

Die präoperative Gastroskopie dient einerseits dazu, die individuell richtige Operationsstrategie zu finden (z. B. Y‑Roux-Magen-Bypass bei Ösophagitis oder Barrett-Ösophagus), und andererseits dazu, bei Bedarf eine präoperative Therapie (z. B. Eradikation von *Helicobacter pylori*) einzuleiten. Eine präoperative Eradikation senkt das Risiko von Entzündungen der Anastomose und des Pouches. Gastroskopisch diagnostizierte Zwerchfellhernien werden standardmäßig im Rahmen des bariatrischen/metabolischen Eingriffs exploriert und ggf. mittels Hiatoplastik saniert [29].

## Optionale Untersuchungen vor bariatrischer Operation

### 24-h-pH-Metrie und Manometrie

Durch 24-h-pH-Metrie und Manometrie können eine Motilitätsstörung bzw. eine abnorme Säurebelastung des Ösophagus diagnostiziert und dadurch ggf. bariatrische Operationsverfahren angepasst werden (Tab. 3). Dies ist besonders bei Refluxerkrankungen, Voroperationen oder Erkrankungen von Magen und Speiseröhre von Bedeutung [30, 31].

### Knochengesundheit/Knochendichtemessung

Die Knochendichtemessung kann bei Menschen mit Adipositas aufgrund des Überschusses an Fettgewebe ungenau sein. Des Weiteren ist das Frakturrisiko bei Menschen nach einer bariatrischen Operation auf mehr als das 2fache erhöht. Um die Knochengesundheit postoperativ bestmöglich zu erhalten, kann präoperativ eine Knochendichtemessung zur Evaluation einer Beeinträchtigung der Knochenstabilität durchgeführt werden. Außerdem wird empfohlen, knochenrelevante Parameter (Kalzium, Parathormon, Albumin, Vitamin D) präoperativ zu messen, um präoperative Mängel von Elektrolyten und Vitaminen, wenn notwendig, schon im Vorfeld zu substituieren [32].

### Alkoholkonsum

Der AUDIT-C-Score [33] ist ein Screening-Instrument, das im Rahmen des World Health Organization Collaborative Project on the Detection and Management of Alcohol-related Problems in Primary Health Care entwickelt wurde und verwendet werden kann, um gefährlichen oder schädlichen Alkoholkonsum präoperativ zu detektieren. Der Score besteht aus 3 Fragen, nimmt nicht viel Zeit in Anspruch und löst in der Regel keinen Widerstand bei den Befragten aus.

## Adipositas-Board

Komplexe PatientInnen mit speziellen Indikationen zur metabolischen Chirurgie oder Reoperation sowie erhöhtem allgemeinem Risiko sollten in einem interdisziplinären Adipositas-Board besprochen werden. Dieses Board besteht im Allgemeinen aus ChirurgInnen, InternistInnen, DiätologInnen, AnästhesistInnen und bei Bedarf weiteren assoziierten Berufsgruppen und sollte regelmäßig tagen.

## Kommunikation mit PatientInnen und mit anderem medizinischen Fachpersonal

Bariatrische Chirurgie ist derzeit die effizienteste Therapieform bei höhergradiger Adipositas, wird jedoch den PatientInnen nach wie vor viel zu wenig angeboten. Begrenzte Kenntnisse über die Wirksamkeit der bariatrischen Chirurgie sowie Sicherheitsbedenken bei PatientInnen, zuweisenden ÄrztInnen und Angehörigen des medizinischen Fachpersonals – trotz exzellenter Datenlage – sind ein Haupthindernis für die Inanspruchnahme der bariatrischen Chirurgie [34]. Weitere Einflüsse sind Vorurteile und Stigmatisierung gegenüber Menschen mit Adipositas und eine suboptimale Kommunikation zwischen PatientInnen und ÄrztInnen. Frauen repräsentieren mehr als zwei Drittel des PatientInnenkollektivs, jedoch haben Männer zum Zeitpunkt der Operation häufiger schwerwiegendere Erkrankungsverläufe, was auch die Mortalitätsdaten widerspiegeln [35].

Eine verbesserte Kommunikation zwischen ÄrztInnen und PatientInnen sowie zwischen medizinischem Personal (ZuweiserInnen, ChirurgInnen) hinsichtlich Risiken, Verlauf und postoperativer Erwartungen sind in diesem Kontext unbedingt zu empfehlen.

Informationsgespräche bezüglich der Zuweisung zur bariatrischen Operation sollten grundsätzlich alle ÄrztInnen, die PatientInnen mit Adipositas behandeln, sowie DiätologInnen, Selbsthilfegruppen etc. durchführen.
